# Lifetime trauma, mental well-being, alcohol and help-seeking; the phenomenological experience of veterans residing in Northern Ireland

**DOI:** 10.1186/s40359-024-01978-1

**Published:** 2024-09-10

**Authors:** Catherine Hitch, Paul Toner, Hannah Champion, Cherie Armour

**Affiliations:** 1 School of Psychology, Queen’s University Belfast, David Keir Building, 18-30 Malone Road, Belfast, BT9 5BN Northern Ireland; 2 School of Psychology, Arden University, Arden House, Middlemarch Park, Coventry, CV3 UK; 3 4FJSchool of Psychology, Swansea University, Singleton Park Campus, Swansea, SA2 8PP UK

**Keywords:** Veterans, Northern Ireland, Experiences, Help-seeking, Mental Health, Alcohol

## Abstract

**Background:**

Veteran residents in Northern Ireland (NI) are an under-researched population. Little is known about their experiences of trauma and mental health management. The overall mental well-being of veterans living in NI may be poorer than other veteran populations, due to the challenges presented by the unique landscape. Understanding their experiences is crucial for providing appropriate, targeted support.

**Method:**

Six male veterans, who had received a mental health diagnosis, living in NI and all aged > 40 years participated. Semi-structured interviews, using open-ended questions, were conducted over the telephone. Interpretative phenomenological analysis was used to explore their experiences.

**Results:**

Two experiential themes were identified each containing three experiential statements. Statements for ‘an extreme lack of’ included: lack of mental health literacy/awareness; lack of expectations of official support; lack of a sense of perceived appreciation. Statements for ‘an extreme abundance of’ included: exacerbated exposure to a range of extreme environments; high levels of ruled-based living; high levels of engaging with informal/local level support.

**Conclusions:**

Several experiential statements aligned with existing literature, including having poor mental health literacy and problem recognition, and heavily utilising social support versus formal help-seeking. Some novel findings included bouncing between extreme positive and negative environments which could be as detrimental to mental health as experiencing conflict trauma. Heavy alcohol use was just another rule soldiers followed. Positive help-seeking experiences failed to improve poor opinions of support organisations. Finally, poor self-perceptions connected to military status are pertinent in NI, which seems to fuel self-marginalisation and distrust. A combination of factors likely contributes to many veterans living in NI having poorer mental well-being. Novel findings would benefit from further exploration as understanding how NI veterans interpret their experiences is key to providing adequate healthcare.

**Supplementary Information:**

The online version contains supplementary material available at 10.1186/s40359-024-01978-1.

## Introduction

Military veterans residing in Northern Ireland (NI) are an under-researched population, mainly due to security concerns connected to the Troubles’ legacy. For context, the Troubles was a period of civil violence that escalated in the late 1960s across NI. It was linked to the deep-rooted, sectarian conflict between those aligned with the British Crown/Protestant identity and those identifying as Nationalists/Catholics [1]. Generally, anyone in NI who is an ex-Crown employee is vulnerable to sectarian violence [1, 2]. To foster a sense of safety it is common for veterans in NI not to disclose their veteran status or previous work-related experiences [3, 4]. This need for non-disclosure and hypervigilance towards security applies not only to native NI veterans but also those veterans who choose to settle in NI at a later date.

Despite the experiences of veterans residing in NI being under-explored, it is fair to assume they have been exposed to elevated levels of lifetime trauma. Moreover, NI veteran preliminary evidence indicates that mental health difficulty rates are equal to, or higher than, a clinical, help-seeking sample [5].

Native NI veterans of a particular age were likely ‘Children of Troubles’ and witnessed localised conflict trauma daily. Moreover, NI has historically experienced high rates of social deprivation, causing a greater likelihood of more adverse childhood experiences [6]. Some NI residents chose to join the local military regiment; the Ulster Defence Regiment (UDR) - to partake in the Troubles. As the UDR were local community members they were living in their own theatre of war. For the UDR, escaping Troubles-related trauma exposure was not possible, even during their leisure time [2] and members of the UDR were often the target of sectarian attacks. They were easier to target as they did not live in secure barracks [2]. Even if an NI native chose to join the military and left NI, particularly during those turbulent times, they could have been deployed to another conflict location [7].

Research regarding elevated trauma exposure has referred to trauma being layered across a person’s life course [8], and having detrimental effects on mental well-being. Literature pertaining to the effect of ‘everyday life during The Troubles’ on mental well-being, found that the fear of trauma exposure had the same negative impact on mental well-being as experiencing actual trauma [9]. If specific traumas experienced by soldiers/veterans are associated with poorer mental well-being [10, 11], it follows that layered trauma (actual and perceived) is likely to be especially detrimental. It appears that veterans residing in NI, especially the former UDR, have experienced a great deal of layered, lifetime, actual or perceived trauma, and are particularly vulnerable to experiencing mental health problems [5].

Military population evidence does suggest there is a historical trend in using alcohol to cope with the psychological effects of (actual and perceived) traumatic experiences [12]. There is also a reported relationship between alcohol difficulties, other co-occurring mental health problems (including PTSD) and summative trauma for veterans in NI [13, 14]. However, alcohol also appears to be linked to non-help-seeking in military populations [12]. This is perhaps because if a soldier was perceived as having poor mental well-being they were considered weak and unfit for purpose, thus poor mental well-being and help-seeking were stigmatised [15]. Instead, soldiers were encouraged to be stoic while the military simultaneously facilitated alcohol use to reduce the need to seek help [12, 16].

Lower help-seeking tendencies often cascade into veteran life, which could partially account for low global help-seeking rates in veterans and higher levels of reported alcohol difficulties [11, 17]. It is perhaps unsurprising that Combat Stress, the UK’s leading veteran mental health charity, reported that veterans tend to present with multiple, co-occurring (complex) mental health conditions, with 95.6% screening likely for hazardous alcohol use. Moreover, Combat Stress found that NI veterans represented a disproportionately large number of mental health treatment attendees and waited an average of 13 years to help-seek [11]. Veterans who delay help-seeking, and spend a protracted time managing their difficulties with alcohol, ultimately do help-seek out of the heightened functional impairment associated with their complex difficulties [18]. Yet, at this stage, their difficulties can be more challenging to treat [18].

If veterans in NI did wish to help-seek, regardless of stigmatised attitudes, resources are particularly low – low for veterans and the general population. The lack of accessible and available resources is also considered to be connected to the Troubles legacy and may be an added barrier to help-seeking for this population [19]. If a veteran has specific healthcare needs, they are generally directed towards the same healthcare pathway as the general population. Statutory, clinical support is limited for all NI residents and waiting lists are lengthy [20]. Regarding the charity sector, increasing numbers of veteran-friendly support groups are appearing in NI and are encouraged to advertise through the NI Veterans’ Support Office (VSO) [21]. However, it is unclear if veterans have regular contact with the VSO, if they understand exactly what each organisation offers support for, or whether healthcare professionals operate within those organisations. Limited or inaccessible resources, together with poor experiences of attempting to use such resources, are key barriers to help-seeking for veterans [22, 23].

To improve or develop services for veterans resident in NI it is essential that their experiences are understood regarding the pathway between trauma and help-seeking, which factors the unique challenges that exist in the NI landscape. Yet, the actual experiences of veterans regarding their response to trauma, and how they manage their mental well-being in the NI context, are unexplored and currently unknown. This study aimed to explore the experiences of any veterans residing in NI in relation to lifetime trauma, mental well-being, alcohol and help-seeking.

## Methods

### Design

To explore the subjective experiences of veterans resident in NI the study adopted a qualitative, phenomenological approach; interpretative phenomenological analysis (IPA) [24]. The IPA approach was adopted to qualitatively explore people’s own real-world, lived experiences. IPA draws on concepts linked to phenomenology, hermeneutics and ideography [24, 25]. There is an acknowledgement within phenomenological approaches that each person’s experience is unique, thus people may converge on their experiences and describe similarities but also have experiential divergent uniqueness. Understanding such uniqueness and sharedness is useful from the perspective of improving services and the help-seeking experiences of specific populations. It was considered that IPA was the most appropriate methodology for this study [24].

This study received ethical approval from the Engineering and Physical Science Research Ethics Committee, Queen’s University, Belfast (EPS 19_286).

### Participants

Eligibility criteria for this study included being = > 18 years, resident in NI (native and non-native to NI) and had sought help for mental health difficulties (which included hazardous alcohol use). Sources of support included statutory support, private care or charities. Types of support were medical, therapeutic or practical care. Exclusions included informal support, such as friends or family. Eligibility was restricted to the definition of a UK veteran: having served at least one day in military service [26]. UK veteran status is not associated with operational deployment. Seven male participants, all aged > 40, provided an interview, but one immediately withdrew their data over security concerns. Having a co-occurring (complex) mental health difficulty was not an edibility criterion, yet all six participants mentioned either alcohol or alcohol and drug-related difficulties as accompanying other mental health problem. The participants presented as having complex mental health difficulties, without reporting they had been diagnosed as such. See Table 1 for participant information. Six participants were deemed an acceptable sample number for this IPA study, given an in-depth analysis was being conducted [24].

**Table 1:** Demographic information

No of participants (*n*)	6*
***Gender***	
Male	6
Female	
***Age***	
< 40	
51–60	2
61–70	3
< 80	
***Highest education level attained***	
Primary school	
CSE/GCSE.	1
O/A level	1
Vocational qualifications	3
Degree	
Postgraduate	
***Employment status***	
Employed	3
Retired	1
Medically retired	2
Unemployed	
***Religion***	
Protestant	5
Catholic	
Other	
None	1
***Length of service in the military***	
< 5yrs	
5-10yrs	1
11-15yrs	2
16-20yrs	
> 20yrs	2
***Length of service in NI***	
< 5yrs	3
5-10yrs	
11-15yrs	2
16-20yrs	
> 20yrs	1
***Relationship status***	
Single	
Married	3
Cohabiting	
Divorced	2
Widow/widowed	
***Have you received an official mental health diagnosis from a health professional*** – *what was it? (e.g.*,* Depression*,* Anxiety*,* PTSD*,* Social Phobia*,* Alcohol*,* Drugs)*	
Yes	6 (3 PTSD, 2 anxiety, 1 did not say)
***Have you had difficulty with any of the above that you did not seek help for***,*** or did not think you needed help for?***	
Yes	6 (5 alcohol, 1 alcohol and drugs)

*one participant chose not to answer all questions, so some of the table is based on responses of five participants

### Recruitment

Participants were recruited via a range of methods. An advert was circulated on social media (Twitter; Facebook) and sent to personal network contacts within specific charities/support organisations (BLESMA veteran limbless charity; Sea, Sailor, Army & Families Association (SSAFA); Combat Stress veteran mental health charity; Royal British Legion (RBL); the Ulster Defence Regiment Aftercare Service; and Alcoholics Anonymous NI. Moreover, an interview was given to British Forces Posted Overseas (BFPO) UK radio to advertise the study. Snowball sampling was encouraged by any participant who agreed to take part.

### Data collection

Participants were provided verbal information, followed by a participation information sheet and consent form via email. Participants were invited to ask questions before partaking. Informed consent was collected verbally over the telephone ahead of each interview.

Single-person interviews were conducted via telephone. Telephone interviews were utilised due to Covid-19 restrictions, however, it was not considered that telephone interviews would compromise the quality of data collection. Studies have found little difference in data quality outcomes between telephone and in-person interviews [27]. Given the veteran population residing in NI was considered hard-to-reach or hidden some may have felt more comfortable participating by telephone due to enhanced anonymity.

Interviews were semi-structured and loosely followed an interview guide, based on open-ended questions. The interview guide included questions relating to participants’ positive and negative experiences concerning living in NI as a veteran, the military, and help-seeking. Veterans were also asked about alcohol use, mental well-being, NI community and culture, identity and perception changes over time. The bespoke interview guide was created by the research team. Care was taken so the questions were not considered so sensitive regarding mental well-being, military service and living in NI that the participants would choose not to answer. For example, potentially ‘incriminating’ or morally injurious questions were not included (see supplementary file).

Interviews lasted from 37 to 134 min and took place between the 11th of May and the 16th of September 2020. Upon interview commencement demographic information was collected; one participant provided partial information to protect their anonymity. All interviews were audio-recorded and transcribed verbatim. Any identifying features were removed entirely or replaced with pseudonyms in the transcripts, to protect anonymity. All transcripts were sent to participants to ensure they found the accuracy and anonymity acceptable. All participants were emailed a £20 Amazon e-voucher code immediately after their interview. Finally, each participant received a verbal and emailed debrief.

### Analysis

Conducting IPA is a multi-stage, inductive process, which elicits emerging themes (known as experiential statements) that are closely linked to the data. Experiential statements are developed during the IPA process, as opposed to being pre-defined and then mined within the data. All transcripts were read line-by-line a few times to become familiar with the participants’ accounts. Salient quotes were highlighted across each transcript, initial notes relating to the quotes were made, codes were assigned to pieces of text, and initial emerging themes (experiential statements) relating to similar codes were created. The next phase involved considering a list of preliminary experiential statements to ascertain if clusters or groups of coded statements sharing a similarity, across all transcripts, could be identified. The list was concluded once inductive thematic saturation was reached; no new codes or experiential statements were elicited [28]. The list of experiential statements was refined; some statements sharing similarities were collapsed into larger, more robust experiential statements. The final list of statements was considered as to whether they were overarching, personal experiential themes or experiential statements. Experiential statements and personal experiential themes were compared against the quotes to ensure the statements and experiential themes were elicited from the data and labelled appropriately [24, 29]. See Table 2 for the final list of experiential statements and personal experiential themes.

### Credibility and trustworthiness

Steps were taken to foster trustworthiness and credibility of the findings of this study. As the experiences of the participants were understood through the interpretation of the research team [29], it is essential to note that one researcher had a former affiliation with the veteran charity SSAFA. During post-interview, peer debrief sessions a discussion was had so that preconceptions and assumptions could be reflected on and considered within the overall data interpretation/analytic process [30]. During the analytic process, CH and PT discussed the formation of experiential statements and personal experiential themes. An expert, external adviser (BH), was utilised to cross-check statements and themes with the transcripts and confirmed appropriate statement/theme labels were formulated. PT and CH reverted to CA, for a final decision, in the event of a disagreement.

## Results

Analysis identified three experiential statements embedded within two personal experiential themes. See Table 2 for the statements and personal experiential themes identified across the six transcripts.

**Table 2:**
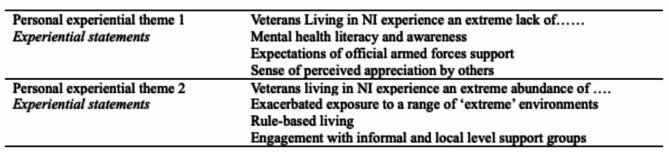
Summary of experiential statements and personal experiential themes

### Personal experiential theme 1: veterans living in NI experience an extreme lack of …

The first personal experiential theme related to participants’ experiencing an extreme lack of something across their lives.

#### Mental health literacy and awareness

The participants generally thought there was nothing wrong with them mentally; they did not believe they needed help despite being prompted to help-seek by others. It was common for participants to repeat the phrase ‘without realising it’, which highlighted their reflection they did have underlying mental health difficulties. There was a tendency to use avoidance, repression and compartmentalisation, which was ultimately unhelpful. Having a lack of awareness/literacy around managing mental health difficulties, and turning to maladaptive strategies, often culminated in reaching a rock-bottom place, such as suicide attempts. Moreover, suicide attempts were frequently multiple, therefore, the first attempt did not seem to lead to an awareness they needed to reach out for support. The lack of awareness extended to not knowing how or where to access mental health support; some did not help seek until it was “thrust into my face” (P1) by a trusted comrade.

I didn’t realise it [I needed help]…she [my wife] suggested I get some counselling and I was always like ‘what for? But you can only deal with so much and the compartmentalising is actually hurting you…[I] just got deeper into a darker place…[after several suicide attempts] I needed to do something to avoid me acting on the urge again. (P3)

#### Expectations of official armed forces support

Participants had low expectations of support linked to the wider government, statutory and health services, and official veteran charities. Experiences that fuelled low expectations often spanned back to active service or occurred as soldiers transitioned out of the military. One veteran described witnessing another soldier’s experience of help-seeking for mental health difficulties as “walking down an alleyway towards a clown with an axe and severed head” (P1). Alternatively, there seemed to be no support at all, which contributed to worsening mental health.

When I was discharged, they closed the door in my face; that was my support [from the MOD]. I was depressed, in pain, anxious, all sorts. I felt suicidal. I had lost everything. (P4)

When participants did help-seek, now as veterans, they often had poor experiences which led to making swift judgements regarding the usefulness of support and the competence of professionals. Within the context of accessing psychological support (via a GP), therapy sessions were either not sufficient (type or duration), participants often felt misunderstood, or they did not like the therapy/therapist. In NI, therapy is mainly outsourced to a third party if accessed via a GP.

My GP recommended something outside the NHS…without it I don’t know what would have happened. If I had to wait six months I wouldn’t be here. I’m lucky I can pay the £40 per session; many can’t. (P3)

I didn’t like my (out-sourced) therapist; we just didn’t gel, but I thought I would give it a go as it was all that was on offer. It [EMDR] didn’t work. I hated it! And then it was cut short anyway. Funding apparently. (P4)

Other poor experiences were connected to accessing support within NI-based charities, where veterans reported having their needs ignored.

Every single charity almost without exception has a tick-sheet. They stick your name down, Mr Smith, did we see him? Tick. Was he contacted in 72 h? Tick. Did we offer something? Tick. Anything we can do? No. Tick. Finished. (P5)

Negativity towards charities was compounded by assumptions that superior support was offered in England. The veterans were aware there were fewer resources in NI and described instances of being offered care overseas. However, travel overseas was not always possible due to mental or physical impairments. It was considered that those who offered such support to the veterans resident in NI did not fully appreciate NI veterans’ needs or that veterans are “fairly dispersed and hiding” (P1).

I’ve a friend who’s originally from NI…he’s utilising [England-based care]… it’s very good. He is definitely being looked after better across there. (P6)

Regardless of what support is offered, and who provides it, there were also those participants who described not trusting anyone (e.g., doctors; housing officers; social workers) due to the perception that workers in certain roles or organisations have sectarian beliefs. Those who reported having these fears described having the lowest expectations of official support, and often made the swiftest negative pre-judgements.

It’s hard because as soon as you go in, and the doctor is from the other [sectarian] ‘side’, you know he doesn’t give a f***. (P2)

There were two accounts of some positive help-seeking experiences. Participants reported resonating with their therapists and that the process was considered beneficial. Yet, these positive instances often followed a series of negative experiences, and the overall perceptions of support in NI remained low.

#### Sense of perceived appreciation

A strong theme expressed was that the NI veterans did not feel appreciated, which may have begun at the outset of military life. During military service, it did not seem to matter what you achieved, as a shadow would be cast over your achievements if you were physically or mentally impaired in some way. This mentality drove many to strive for perfection, to be respected and appreciated.

Imagine I am the battalion shooting champion; I’m the cross-country champion, I play football, I play golf and I’m always in the top tier… represent the military and flies the flag…the day I break my leg, and I must go sick they don’t remember the sportsman, the good representative; it’s that waster [they remember]. (P4)

This sense of being undervalued continues into veteran life. Some felt that official support organisations failed to demonstrate appreciation for the veteran community, and inappropriately took credit for their positive actions and initiatives.

The saddest thing is there’s loads of veterans who are willing to give up their time but they’re not going to give it to an organisation, or on behalf of an organisation, that’s going to take the credit for it. (P5)

The other source of non-appreciation experienced acutely by many participants was linked to the general NI public. In contrast to US veterans, they felt their service was not recognised nor valued as it should be, which contributed to the sense of self-marginalisation within NI.

When I joined ….my belief would have been that when I completed my service I would not be praised or acknowledged….it’s not just the government but a huge section of the British people. I feel they don’t acknowledge the sacrifice that servicemen and women have done. In comparison, the US are a pretty proud [of the military] public. Definitely makes me want to hide my status. (P5)

### Personal experiential theme 2: veterans living in NI experience an extreme abundance of …

The second personal experiential theme related to participants’ experiencing an extreme abundance of ‘something’ across their lives.

#### Exacerbated exposure to a range of ‘extreme’ environments

Participants described being embedded within environments containing what could be conceptualised as ‘extremes’. Considering the span of a veteran’s life course, particularly within NI, the first extremes encountered were likely related to local trauma and violence. Some participants inferred they had grown up as ‘Children of the Troubles’. Participants who chose to join the UDR often experienced direct Troubles violence on their local streets. Non-natives who were deployed to NI also experienced Troubles violence. Members of the security forces situated in NI were generally the direct target of sectarian violence, with two participants describing themselves as being the ‘mark’ of violent attacks. Moreover, many participants also experienced military-related trauma outside of NI, not limited to operational exercises.

The lows that really stay with you are the tragedies, people died … the easiest one [to recall] was when a young fellow died on [foreign] exercise. It was tragic. He died in a training accident and not even on tour. (P1)

Participants who chose to settle or remain in NI after military service were transitioning as veterans within an environment they generally perceived as traumatic. Living with the threat of injury or death directed at Crown affiliates brought a sense of extreme hypervigilance and distrust of outsiders, and participants were managing a constant feeling of animosity. P2 described it as constantly being a legitimate target for violence because “this population, they hate you and want to kill you”.

Native veterans seemed to have travelled a full ‘trauma-exposure circle’; they had been born into traumatic exposure, continued to be exposed to environmental extremes through military life, and had retired into an environment with perceived or actual threat of trauma.

I was raised and witnessed a lot, the discontent, the rioting. [From home] I watched gun battles, I used to hear gun battles, I used to see hooded men walking about the streets…[while in the UDR] a person set me up to get killed, and then again in the [regular army] service. (P5)

Aside from experiencing environmental trauma, some described engaging in activities at an extreme level, including playing sports and partying. Not only did veterans describe shifting between these environmental extremes (traumatic and pleasurable) regularly, but sometimes it occurred quite suddenly.

When you stop [while on deployment or training], it’s like you’re catching up on six weeks of living in a hole. Fitting six weeks life into a two-week gap before you do it again. Everyone does it…you try to live two days in one….you are trying to put the days you have lost socially into the days you have got so you go further; double up; you party harder before going off again. (P1)

[When not on tour] I decided to do an Iron Man for my first triathlon and not a basic triathlon…I was encouraged to compete for the UK. ….I ran 115 miles in 26 h [in a separate ultra running event] and placed within the top 15 of the whole UK. (P2)

Given that the veterans described transitioning between combat deployments, sometimes without decompression, and experienced periods of intense partying or ‘activities’, they seemed to regularly be in some kind of ‘extreme’ state or situation (physically and mentally).

#### Rule-based living

Much of military life was described as heavily ‘rule’ or procedure based. There was unquestioning respect for the rules, which were strict and inflexible and instilled from the outset of military life. Rule-breaking was stigmatised and deviating from the rules (e.g., formally help-seeking for mental health difficulties) could be harmful to one’s career.

We weren’t even allowed to go home for the funeral … you get on with it. You get over it. It was the policy. You don’t go to counselling or anything if you want your career; you just crack on. (P3)

Instead of help-seeking via therapy to manage stress and trauma, it seemed alcohol was used excessively. Alcohol use was both encouraged and permitted as self-medication, to ultimately help soldiers get back to work.

During service, there was an awful lot of alcohol and a lot of sadness… he died we came back to camp, we packed up his stuff, we had a funeral, we got drunk, I was sad, we went back to work. (P1)

Alcohol was also used to aid socialisation and celebration. It extended beyond its being a permitted substance and instead was expected and ritualistic. Drinking excessively seemed to be a cultural rule, with alienation being a consequence of non-conformity.

You go into the mess and you’re heavily encouraged to drink before you go in and sit down around seven. You’ll not leave the table. You are not allowed. Waiters, waitresses come and fill your glasses with wine and port. You don’t get an opportunity to say, ‘I can’t drink wine; could you get me a Fanta orange’… if you didn’t join in, you were alienated. (P5)

Once some left the military, they appeared to change their alcohol-related attitudes and behaviours. Participants often realised that holding such rule-based attitudes/behaviours is unnecessary and maladaptive, and chose to no longer engage with the overuse (or sometimes any use) of alcohol.

A lot of guys [veterans] want to go for a coffee you know, and a chat [rather than the pub]…I would avoid places like the Legion as I was trying to be different; I just didn’t want to be around that [the drinking culture]. I wasn’t me anymore and doesn’t help me. (P4)

Similarly, the need to challenge and break the no-help-seeking rule was acknowledged and encouraged by many participants, usually due to poor quality of life and functional impairment. They reasoned that although as soldiers they did not break that rule, it no longer applied once the military ‘connection’ was severed.

While I was serving [I was] incredibly reluctant to [get] help but once I left there was no connection or...[no barrier]. (P1)

#### Engagement with informal and local-level support

Participants expressed having an abundance of informal support through camaraderie and veteran connection. Long-lasting camaraderie often came from sharing intense operational experiences. Veterans considered they were best placed to offer support to each other, due to the peer bond they shared.

Six weeks living in a trench, sharing experiences together means you get very close. It’s hard to replicate the social connection outside the military. (P1)

Most participants commented that they socialised within NI with members of the ex-military or ex-security as they mutually respected each other’s privacy and opinions surrounding security concerns. Although the need to stay vigilant was stated by all participants, most described being friendly with some select community members and having a few close local friends who were non-Crown affiliates. Despite a lack of official, publicised social events in NI, for security reasons, a few select events were well supported by local veterans. Moreover, there were non-veteran-specific membership clubs that enabled veteran interaction, such as the Freemasons or the Orange Order.

Remembrance Day’s a great occasion for it [reconnection] because we all get together. My local Orange Lodge seems to have a disproportionately high number of ex-UDRs. (P1)

Some ‘unofficial’, veteran-specific, often veteran-run, activity clubs existed across NI. The local clubs were engaged with by veterans in a variety of ways; through receiving support, getting involved in local support, or both. P5 described running such a club that facilitated fellowship; it brought people together.

Veterans were invited along, we’d go out, we’d have a bit of lunch and a bit of craic. We started to meet once a month but that went to one day a week, as people wanted to meet more often. (P5).

Another source of local, informal support came from families. Some participants opted to move nearer to their families, who provided them with support and mental health relief.

My family helps me [mentally]…seeing my kids makes my heart glow…seeing my kids is probably the biggest thing [that helps me]. (P2)

## Discussion

This study aimed to explore the experience of lifetime trauma, mental well-being, alcohol use and help-seeking from the perspective of veterans residing within NI. While the two personal experiential themes were presented as ‘lack of’ and ‘abundance of’ they were not polar ends of the same continuum. For example, literacy awareness was only associated with the ‘lack of’ personal experiential theme; there was no opposite experiential statement found under the ‘abundance of’ personal experiential theme.

The first novel finding in this study relates to NI residents likely having elevated levels of extreme experiences or being in extreme environments. ‘Extreme’ in this context refers to experiences or environments that have deviated from what ‘most’ people would consider was ‘normal’ or low risk in everyday life. It is fair to suggest that ‘most people’ do not live with a constant threat of physical and psychological violence linked to war or civil conflict [31]. Nor do they choose a vocation that has higher risks to life or engage in sports to an extreme level, let alone a combination of these. Although existing literature has discussed the impact of soldiers’ combat exposure, operational deployment, and transition to veteran/civilian life [32–35], no literature has addressed the collective and sometimes rapid swing between diverse extreme environments. Such diverse environments may include things perceived as positive (sports; partying) or negative (combat). Swinging between experiences and environments warrants further exploration.

A point regarding environmental extremes that warrants further discussion is the experience of layered, lifetime trauma [8] ranging from childhood to military trauma and continuing onto post-Troubles trauma. Essentially, it may be that veterans residing in NI cannot (currently) escape trauma exposure which makes them (or their environment) unique compared to other UK nations (and most global veteran populations). The degree to which the NI landscape was experienced as particularly traumatising by the participants was a matter of interpretation as living in NI may have led to some resilience towards certain traumas [36, 37]. Although many suggested their most significant traumas related to foreign deployments, the main driver for wanting to remain hidden was the persistent threat of injury or death to themselves or significant others while having a veteran status in NI [3, 4].

While the participants were managing a range of lifetime traumas, the participants had at times been engaging in extreme levels of alcohol-fuelled socialising which aligns with existing evidence [12]. The opinion that alcohol ‘lubricates the military’ is engrained historically and often cascades into veteran life [12, 18, 38]. All participants in this study discussed socialising and drinking heavily, to cope with operational stress and to catch up on the sense of missed recreational time (albeit, more so as soldiers than veterans). Moreover, alcohol was used communally to engage in shared coping, consolation, commemoration, celebration, or for any other reasons the soldiers chose to drink. A novel finding in this study was the degree to which participants described their drinking behaviour as just another form of rule-following. There were consequences if participants did not consume alcohol at arguably ‘extreme’ levels. The military is known for its conditioning through rule compliance, with the British Army’s values and standards emphasising the need for courage and discipline. Extreme alcohol-related behaviour could be explained as the participants doing what was expected, encouraged and facilitated versus acting with any autonomy [35]. If veterans did lack autonomy over their drinking, it begs the question as to how accountable they are for their alcohol-related behaviour. It would be interesting to explore whether levels of perceived or actual autonomy have changed in the military and whether that has had any effect on unhealthy coping behaviours as soldiers and later as veterans. While several participants did challenge opinions on alcohol behaviour, once they had lived as veterans for a while, there may be many veterans who continue to live by military-based rules that cascade into their veteran life.

One shift in current UK military training is intended to reduce alcohol use as an unhealthy coping strategy and lower the risk of developing an alcohol use disorder, as a serving soldier or later as a veteran. OpSmart aims to increase resilience in the context of trauma and reduce forms of maladaptive coping, such as drinking or other risky behaviours [39]. However, the effectiveness of OpSmart is currently unknown. Older veterans, who are more representative of this study participant group (i.e., =>40), likely never received OpSmart training, which may partially account for participants having an extreme lack of mental health literacy.

The extreme lack of mental health literacy found in this study does align with existing evidence [16, 22] and may partly account for no participants reporting they sought formal help for alcohol (or drug) use. Veterans may not be aware that alcohol use is classified as a mental health disorder, depending on the levels of consumption and effects of alcohol-related behaviour [40]. Soldiers may have not considered the dangers of hazardous alcohol use if its consumption was largely facilitated by their employer [12]. Moreover, if alcohol use did not cause the same level of functional impairment compared to, say, PTSD, participants may not have perceived the need to help-seek. A lack of problem recognition in veterans, and the preference for self-care, are both reported across veteran literature [41].

If veterans did want to seek help for concerns over problem alcohol use in NI they could approach Drug and Alcohol NI [42], who provide substance support information for NI residents. This addiction support service operates on a priority ‘tiered’ system, which veterans may experience as inaccessible and overwhelming. Moreover, at the time of writing the website had many broken links which would no doubt act as a further barrier to support seeking.

Regardless of the barriers to support that exist in NI [20], all participants did describe help-seeking out of need, which is not a novel finding [16]. Military literature has generally found approximately 1/3 of global serving and non-serving military populations have reportedly sought help for a mental health difficulty [17]. In this study, the participants appeared to seek help, after a protracted period, and only did so as veterans. They described avoiding help-seeking when they were soldiers as they did not wish to be seen as rule-breaking, despite at least one participant suspecting they ought to seek support for an underlying problem. Taking a protracted length of time to help-seek aligns with Campbell and colleagues’ study, which found NI veterans took on average 15.9 years to access Combat Stress for mental health support [43]. The comments on when to challenge stigmatised help-seeking beliefs and behaviours supported the (novel) rule-following theme. Breaking these rules as a veteran could be interpreted as a form of self-care that was born out of a realised sense of autonomy.

It follows that if veterans in NI experience struggles when accessing support or do not think official support is needed, they are more likely to seek out local-level support. Participants in this study had high levels of local, informal support engagement. Social support does appear to bolster mental well-being and act as a help-seeking enabler for veterans across the veteran literature [16]. Moreover, local support may assist with psychological and practical issues, and foster a sense of belongingness and purpose, which are positive for mental well-being and quality of life outcomes [44–46]. It seems that veterans in NI experienced local support as more accessible, and it met their mental well-being and privacy needs. All the participants explained they formally sought help out of need, which does support existing veteran mental health literature especially if the veterans identify they have a problem and are concerned it will escalate [16, 17, 19, 47–49]. However, a novel finding was that formal help-seeking did not improve participants’ attitudes towards formal support services, even if the mental health outcome was eventually positive. Veteran literature often describes the opposite; attitudes towards support as improving, once trusting relationships with healthcare professionals are built and outcomes are positive [49]. Instead, within this study the opinion that informal, local-level support was valued as optimal was reinforced.

Although participants had a higher level of positive engagement with local, grass-roots support groups, they conversely described having a largely negative relationship with the wider public. The participants described living in an environment that lacked a sense of appreciation towards them as soldiers or veterans. Generally, the lack of appreciation is mirrored across extant global armed forces community literature. Vietnam, Iraq and Afghanistan veterans have had hostility directed towards them due to opinions over those military campaigns [50–52]. Yet, the sense of hostility and disfavour directed at many NI veterans appears to be connected to Crown affiliation and not a particular military campaign. The negativity was reported to feel a lot more personal, especially among those who served locally with the UDR. These findings are concerning because it may lead to worse mental health outcomes and fuel self-marginalisation [50]. It may be the case that the UDR, in particular, have experienced or live with moral injury as they were policing their own streets and witnessed, or took part in, traumatic events involving their neighbours and community. As the negative effects of moral injury can be moderated by a sense of social connectedness, feeling that poor sense of public perception would compound poor mental health [53].

### Limitations and strengths

This study had an underrepresentation of females and officers, which was to be expected as the military is predominantly male, who are non-officers. While generalisation is not the goal of qualitative research, especially studies based on phenomenology, we do acknowledge these findings are not transferable to the wider veteran population in NI.

Detailed information regarding the participants’ overall treatment history was not collected, which could have helped provide a more in-depth understanding of what support was offered and its effectiveness. The study was conducted during Covid-19, and although none of the participants referred to it during their interview, maybe it impacted their experiences and perceptions.

This study did have several strengths; it is the first to qualitatively explore the experiences of veterans (native and non-native) living in NI. Therefore, the findings are novel with respect to this hard-to-reach population. The participants provided open and candid accounts, possibly because they enjoyed the anonymity of taking part over the telephone.

## Implications, suggestions and conclusions

While it is acknowledged these findings do not represent the wider veteran community in NI, it may be the case that many veterans continue to stay hidden, or prefer to use local support organisations, to manage their mental health needs. Although social support is a useful resource it may not be sufficient to manage the mental health difficulties that some veterans in NI have. If adequate resources do not exist in NI, or resources are inaccessible, mental health difficulties would likely be compounded.

Another cause for accessing social support is that grass-roots organisations create a space where veterans feel appreciated and welcomed, and local veterans may perceive they have some control over their own needs. Efforts ought to be made by official organisations and statutory services to engage with the grass-roots organisations, but in a manner that does not cause any veterans to fear for their safety. If official support can reach veterans via such channels, or perhaps through the NI Veterans’ Support Office [21] (VSO – the point of contact for organisations supporting NI veterans) or the newly built Veterans Centre ‘Harbour House’ [54], this may help improve veterans’ opinions of wider NI official support.

Although there is currently no obligation for any doctors’ surgery to be ‘veteran-friendly’ in NI [55], local surgeries could choose to take that stance and advertise themselves accordingly (locally and via the VSO).

If barriers to support cannot be removed, organisations should continue to work collaboratively to increase awareness and education about veterans in the community to reduce ‘them versus us’ thinking. Accessible education around recognising mental health conditions may raise awareness and encourage help-seeking sooner. For clarity, mental health and addiction organisations should provide clear, accessible descriptions of conditions they support (i.e., alcohol, PTSD, complex PTSD, anxiety, depression) and that they are veteran friendly. All websites should be maintained and accessible via a range of mobile devices.

Mental health awareness education could also signpost towards a growing number of veteran-specific smartphone apps. Drinks:Ration is an alcohol reduction app created for UK veterans [56] and the US Veteran Affairs (VA) hosts a number of mental health/mental well-being apps aimed at US veterans, which other nations may benefit from [57]. Apps may provide some relief for the particularly hard-to-reach and act as a stepping stone to more formal support.

As and when veterans do engage with support, and they choose to disclose their veteran status, it would be useful to reflect on the degree to which they are a product of their rule-abiding environment. Acknowledging the effect of rule-based living is important within a therapy support context. Participants in this study demonstrated that some veterans do challenge rule-based thinking, therefore they ought to be empowered and encouraged to do this (adaptively) in a supportive/therapeutic environment.

Wherever possible a Patient Public Involvement (PPI) [58] approach should be adopted, so that NI veterans are included as stakeholders in their own public awareness campaigns and collaborative (organisation/statutory/community) projects. It would be useful to explore through research the outcomes and impact of these sorts of approaches to improving support for veterans living in NI.

## Electronic supplementary material

Below is the link to the electronic supplementary material.

Supplementary Material 1

## Data Availability

The datasets used and/or analysed during the current study are available from the corresponding author on reasonable request. The data has not been deposited publicly due to its sensitive nature.
