# Supplementary material for: Lifetime trauma, mental well-being, alcohol and help-seeking; the phenomenological experience of veterans residing in Northern Ireland
Source: BMC Psychol. 2024 Sep 10;12:479. doi: 10.1186/s40359-024-01978-1 (PMC11386114; doi:10.1186/s40359-024-01978-1)
Supplement: Supplementary file 1 — Supplementary Material 1 [file 40359_2024_1978_MOESM1_ESM.docx]

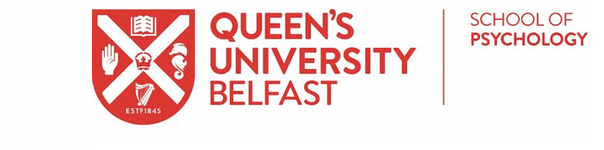


**INTERVIEW SCHEDULE**

1. Can you recall what made you want to join the Armed Forces?

Prompt: was it for financial reasons, does your family have a history of joining the Armed Forces, or did you want to keep your own streets safer during the Troubles?

2. Would you say you have had positive and negative experiences as a soldier and as a veteran?

Prompt: what were those experiences, how have they changed over time? what were your most memorable positive experiences and why; what were your most memorable negative experiences and why: how did you cope with negative experiences over time? Would you say a negative experience was related to trauma as a soldier or as a veteran?

3. Have you in the past or do you currently experience mental health difficulties?

Prompt: would you attribute any such difficulties to your military life, veteran life, a personal event perhaps?

4. What are your views and experiences of help-seeking for mental health issues?

Prompt: Have you ever considered help-seeking for any issues? If you did help-seek, how long did it take for you to recognise that you needed help? What was your experience of help-seeking? Where would you help-seek? Did you find it difficult to access or get help? Were you ever encouraged to help-seek? Who encouraged you to seek help?

5. What would you say your methods of coping are?

Prompt: Are your methods of coping shaped by the military? Is it a ‘man’ thing?

6. Sometimes people use alcohol as a coping mechanism. Why do you think this might be?

Prompt: Do you think this is a cultural thing (male culture or Northern Ireland culture)? Do you think this has a connection to military and veteran attitudes around alcohol?

7. If you suffered mental health issues previously do you think the local community made a difference to your mental health (make it worse or better)? Did you seek out or receive informal sources of support?

Prompt: How did they make a difference? Did the local church make a difference, being part of veteran clubs, other clubs (the RBL, Masons or Orange Lodge), a strong sense of community? Did you receive any help whatsoever from outside the medical profession?

7. Do you think the perception of your experiences has changed over time?

Prompt: what do you think/feel differently now compared to in previous years? What do you think has caused the different perception?

8. Do you identify with the veteran community? Do you identify with the wider community? Do the community know you are a veteran or where you served?

Prompt: What has been your experience of living as a veteran in the Northern Ireland community? Has your identity as a veteran, or as a particular community member, made an impact on help-seeking. Has your identity made you feel more or less inclined to seek help? Has your identity perhaps protected you against trauma?
